# Supplementary material for: Müller glial microRNAs are required for the maintenance of glial homeostasis and retinal architecture
Source: Nat Commun. 2017 Nov 17;8:1603. doi: 10.1038/s41467-017-01624-y (PMC5693933; doi:10.1038/s41467-017-01624-y)
Supplement: Supplementary file 1 — Supplementary Information [file 41467_2017_1624_MOESM1_ESM.pdf]

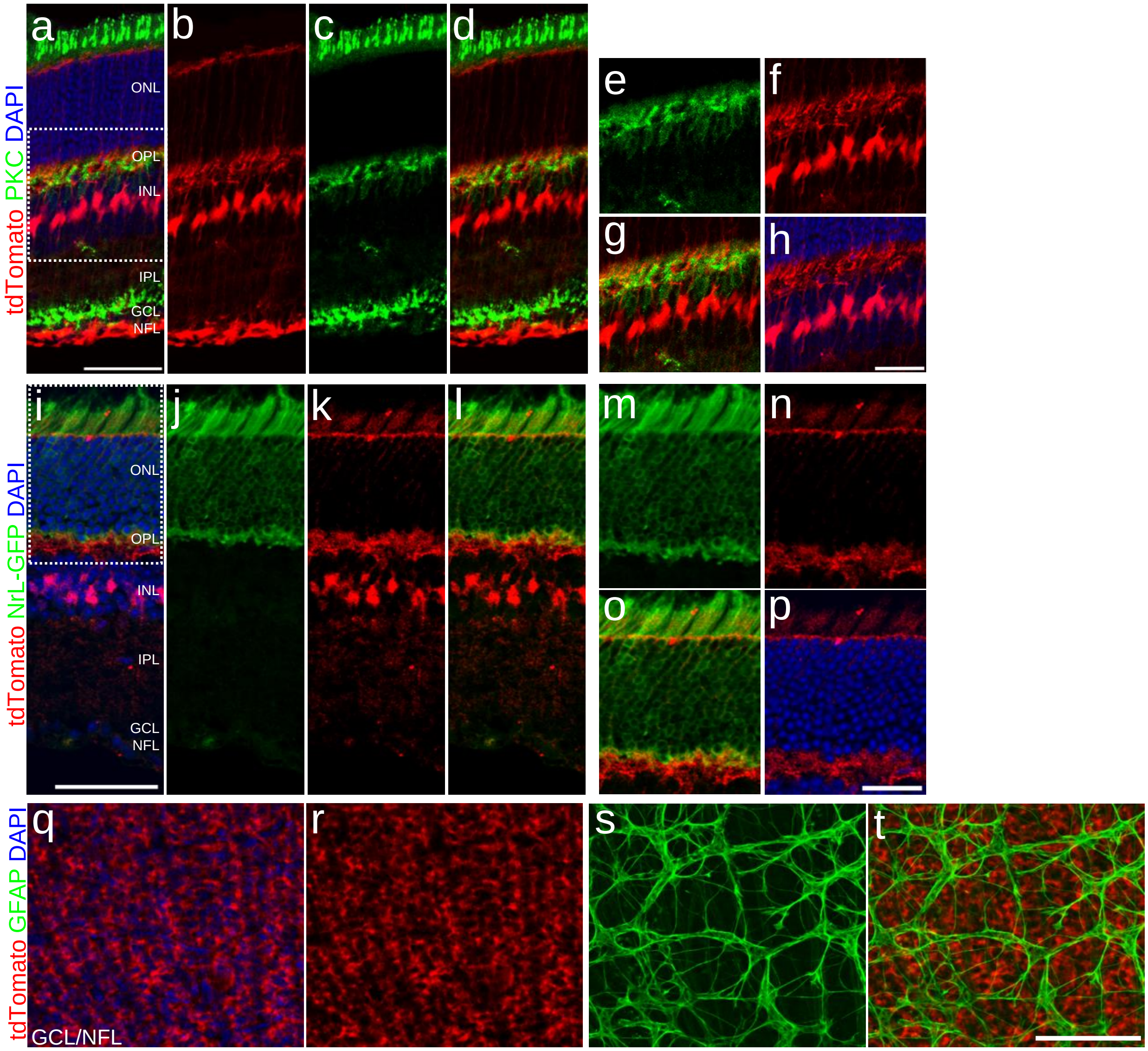

Supplementary Figure 1. Rlbp1:CreER:tdTomato drives reporter expression in Müller glia, not in astrocytes or neurons

Immunofluorescence for tdTomato and the bipolar cell marker PKC (a-h), GFP to label Nrl expressing photoreceptors (i-p), or the astrocyte marker GFAP (q-t) as well as DAPI nuclear staining in an adult wt mouse which received Tamoxifen at postnatal day (P) 11-14 in retinal cross sections (a-p) or flat mount retina (q-t). Scale bars in a, i, t: 50 µm, in h and p: 25 µm. MG: Müller glia, wt: wild type, ONL: outer nuclear layer, OPL: outer plexiform layer, INL: inner nuclear layer, IPL: inner plexiform layer, GCL: ganglion cell layer, NFL: nerve fiber layer.

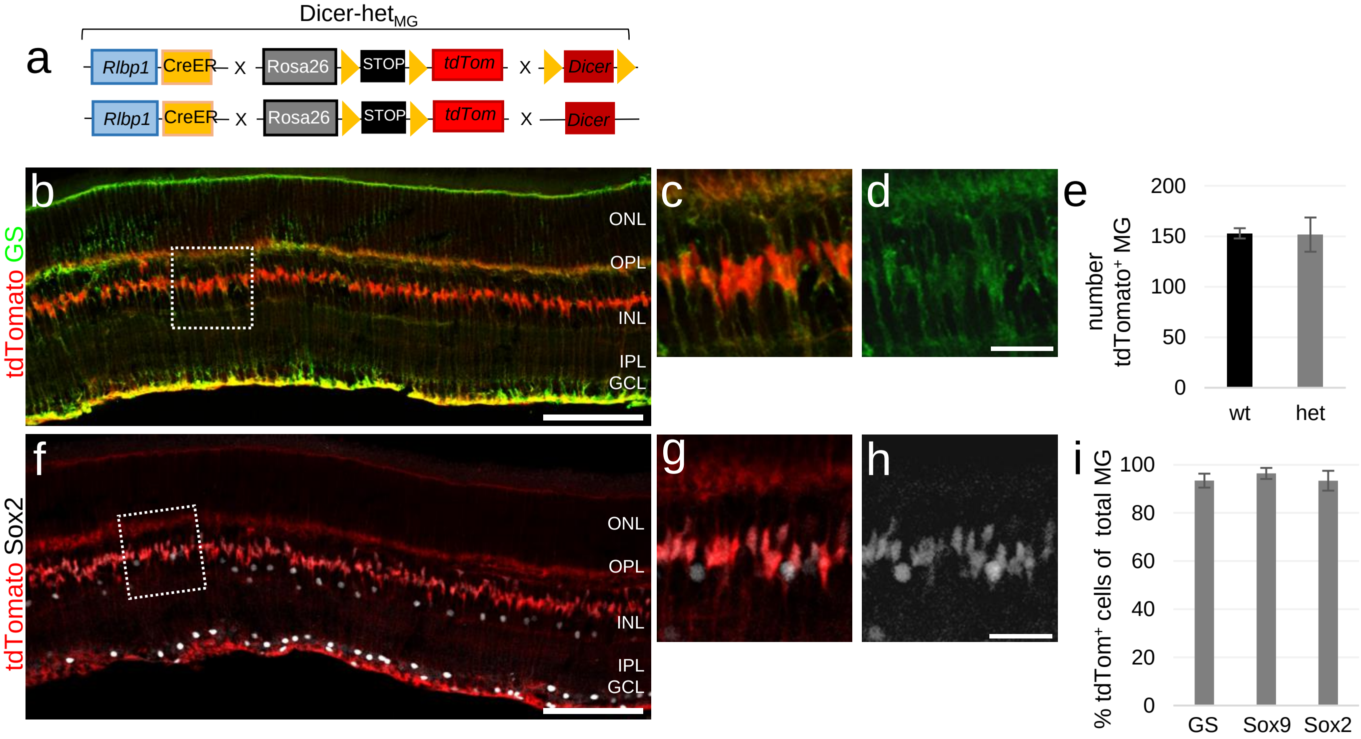

Supplementary Figure 2. Dicer heterozygous mice are not different from wild type mice
a: Schematic of the Dicer1-heterozygous genotype: *Dicer* $^{f/-}$*: Rlbp1CreER: stop*$^{f/f}$*-tdTomato* genotype (Dicer-het$_{MG}$). **b-d, f-h:** Immunofluorescence for tdTomato, Glutamine Synthetase (GS), Sox2, and DAPI nuclear staining of Dicer-het$_{MG}$ retinal cross sections. **e:** Absolute numbers of tdTomato$^+$ MG per field in wild type (wt, n = 5) and Dicer-het$_{MG}$ retinas (n = 5). **i:** Percentage of tdTomato$^+$ MG of total GS, Sox9, or Sox2$^+$ MG in Dicer-het$_{MG}$ retinas. Statistics: mean ± S.D., Independent Samples T-Test and Levene's Test for Equality of Variances, 2-tailed, p (e) = 0.885. Scale bars in b, f: 100 µm, in d, h: 25 µm. MG: Müller glia, wt: wild type, ONL: outer nuclear layer, OPL: outer plexiform layer, INL: inner nuclear layer, IPL: inner plexiform layer, GCL: ganglion cell layer.

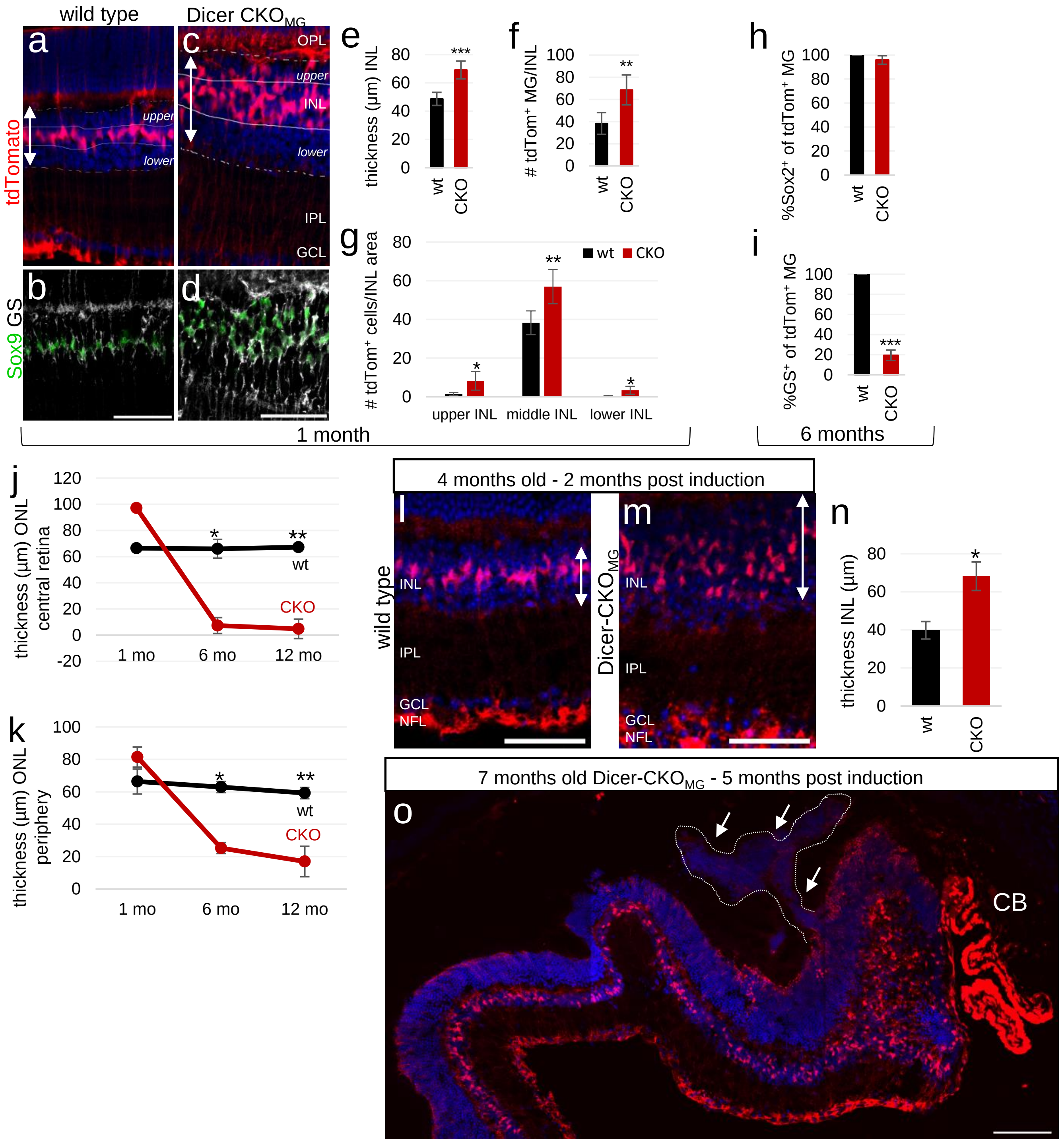

Supplementary Figure 3. Loss of Dicer1 in Müller glia leads to expansion of the INL after deletion in juvenile or adult mice **a-d:** Immunofluorescence for tdTomato, Sox9, Glutamine Synthetase (GS), and DAPI in wt (a, b) and Dicer-CKO$_{MG}$ (c, d). Thin lines mark and divide the INL into an upper, middle, and lower part. **e:** INL thickness measured in wt (n = 6) and Dicer-CKO$_{MG}$ retinas one month after deletion (n = 8,). **f:** Number of tdTomato$^+$ cells per field in the INL of wt (= 6) and Dicer-CKO$_{MG}$ retinas (n = 6). **g:** Number of tdTomato$^+$ MG per field found in the upper, middle, and lower INL in the wt (n = 6) and Dicer-CKO$_{MG}$ (n = 6). **h:** Percentage of tdTomato$^+$ Sox2$^+$ glia of all tdTomato$^+$ cells 6 months after Dicer deletion (wt n = 3, CKO n = 4). **i:** Percentage of tdTomato$^+$ GS$^+$ glia of all tdTomato$^+$ cells 6 months after Dicer deletion (wt n = 3, CKO n = 4). **j-k**: Thickness of the ONL in the central (j) and peripheral retina (k) 1, 6, and 12 months after Dicer deletion in the wild type (wt$_{1mo}$ n = 3, wt$_{6mo}$ n = 4, wt$_{12mo}$ n = 3) and Dicer-CKO$_{MG}$ (CKO$_{1mo}$ n = 4, CKO$_{6mo}$ n = 4, CKO$_{12mo}$ n = 7). **l-m, o:** Immunofluorescence for tdTomato (endogenous unlabeled) and DAPI nuclear labeling of Dicer-CKO$_{MG}$ mice in which Dicer was deleted at postnatal day (P) 56-59 (2 months of age), 2 months after deletion (l-m) or 6 months after deletion (o). **n:** thickness of the INL in wt (n = 3) and Dicer-CKO$_{MG}$ mice (n = 3) 2 months after Dicer deletion. Statistics: mean ± SD, Independent Samples T-Test and Levene's Test for Equality of Variances, 2-tailed, significant differences are indicated, **: $p < 0.01$, ***: $p < 0.0001$. Scale bars in b, d, l, m: 50 µm, in o: 100 µm.

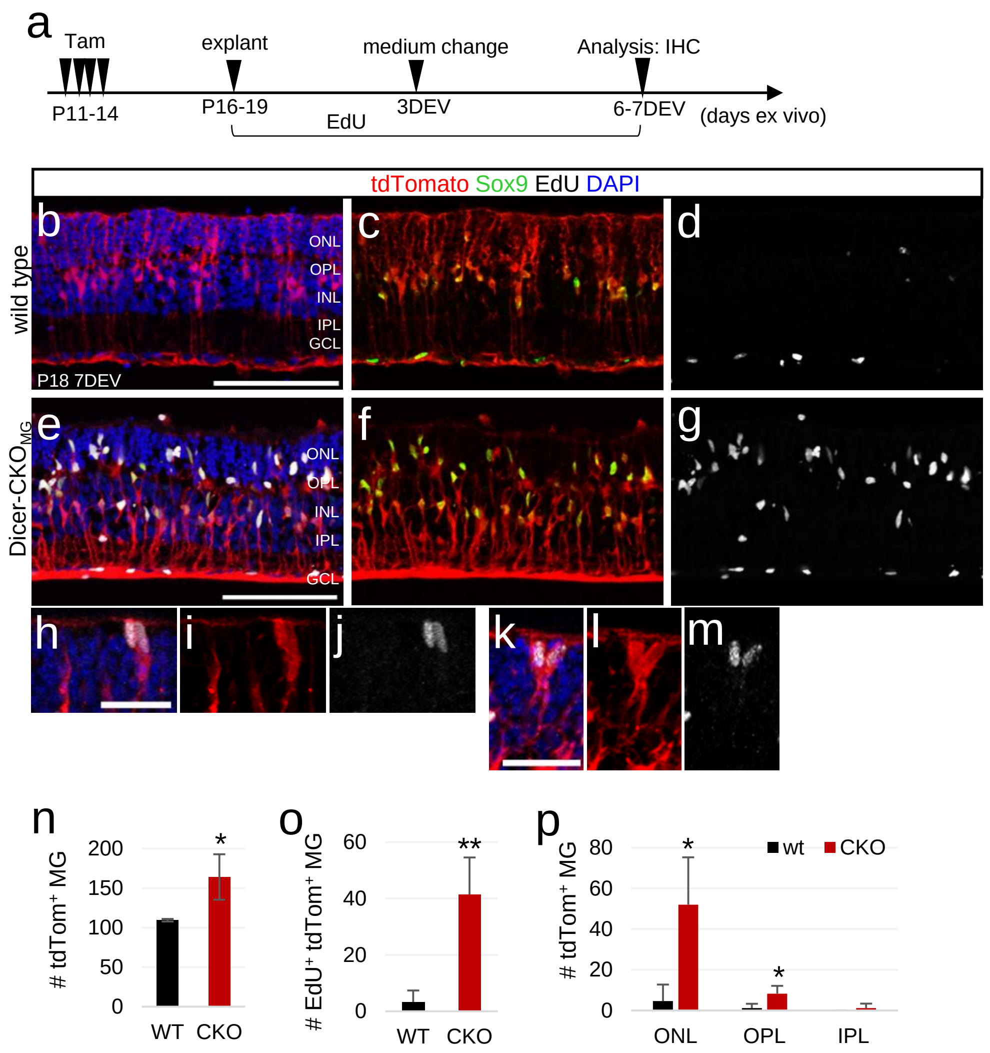

Supplementary Figure 4. Proliferation assessment in Dicer-CKO$_{MG}$ explant cultures

a: Experimental design. **b-m:** Immunofluorescence for tdTomato and Sox9, EdU and DAPI nuclear labeling of P18 wt (b-d) and P18 Dicer-CKO$_{MG}$ explant cultures after 7 days *ex vivo* (DEV, e-g). MG migrate and divide in the ONL (h-m). **n:** Number of tdTomato$^+$ MG per field in wt (n = 3) and Dicer-CKO$_{MG}$ explants (n = 5). **o:** Number of tdTomato$^+$ MG per field in the ONL, OPL, and IPL in wt (n = 3) and Dicer-CKO$_{MG}$ explants (n = 5). **p:** Number of EdU$^+$ tdTomato$^+$ MG per field in wt (n = 3) and Dicer-CKO$_{MG}$ explants (n = 5). Statistics: mean ± S.D., Independent Samples T-Test and Levene's Test for Equality of Variances, 2-tailed, significant differences are indicated, *: $p < 0.05$, **: $p < 0.01$. Scale bars in b, e: 100 µm, in h, k: 25 µm. MG: Müller glia, wt: wild type, ONL: outer nuclear layer, OPL: outer plexiform layer, INL: inner nuclear layer, IPL: inner plexiform layer, GCL: ganglion cell layer.

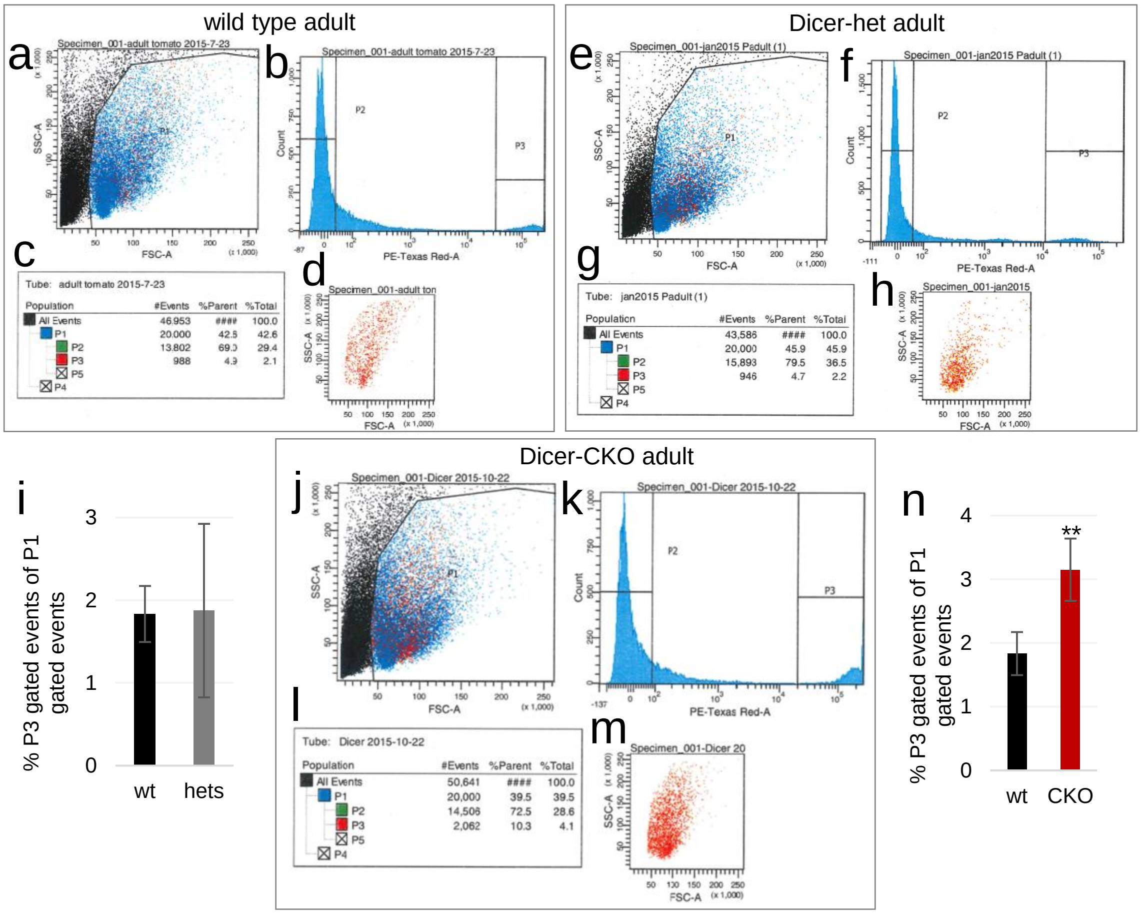

o<br>sample	# sorts	# retinae	# cells in suspension “positives“	postsort purity “positives“	% of total P1 events	% of total events	# cells in suspension “negatives“	postsort purity “negatives“
wild type	6	40	4444839	96%	5%	1.8%	56.5 Mio	0%
Dicer CKO	6	28	5317438	94%	9%	3.3%	34.3 Mio	0%
Dicer het	3	26	2729509	95%	5%	2.2%	20.3 Mio	0%

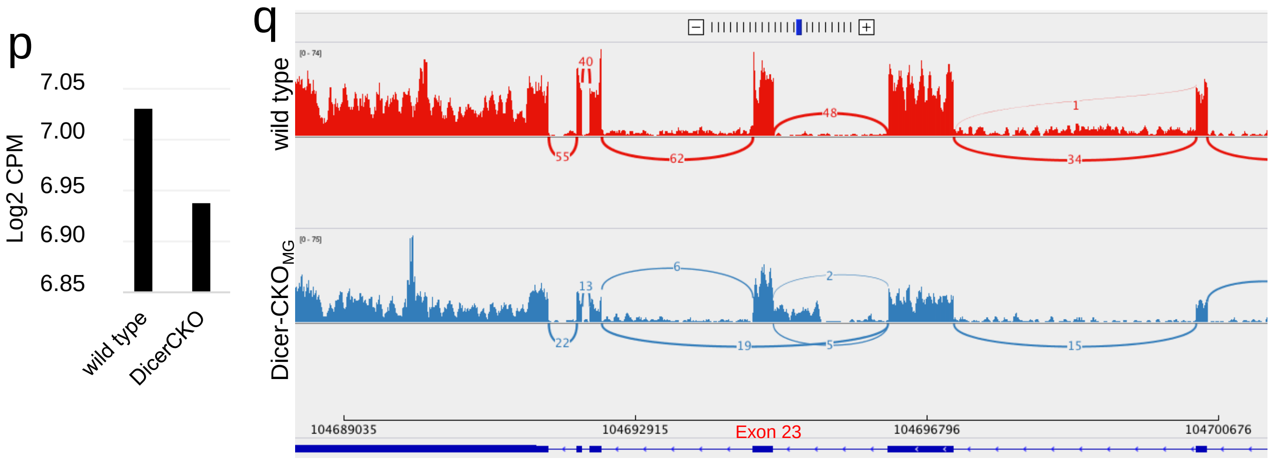

Supplementary Figure 5. Fluorescence-activated cell sorting of Müller glia and assessment of Dicer deletion using RNA-Seq
a-h, j-m: Gating for FACS and proportions of sorted cells. All cells in gate P1 (blue dots) have been sorted based on their size (forward scattered, FSC) and fluorescence/ granularity (side scattered, SSC) for wild type (a-d), Dicer-hets (e-h), and Dicer-CKO (j-m). Very small cells and debris (black dots) have been excluded (a, e, j). tdTomato$^+$ cells were found in gate P3 (brightest fluorescence), while the vast majority of tdTomato$^-$ cells were found in gate P2 (no fluorescence, b, f, k). The percentage of the P2 and P3 events of either P1 or total events or are shown in c-d, g-h, and l-m shows the fraction of the sorted tdTomato$^+$ cells in the SSC and FSC-A scheme. **i:** Percentages of the P3 gated MG fraction of all P1 events, obtained from wild type (n = 6) and Dicer-het$_{MG}$ retinas (n = 4) during fluorescence activated cell sorting (FACS). **n:** Percentages of the P3 gated MG fraction of all P1 events, from wt (n = 6) and Dicer-CKO$_{MG}$ retinas (n = 6) from FACS. **o:** FACS data, input and purity. **p:** schematic of the Log2 of counts per million (CPM) of the overall reads in the whole Dicer gene in the wt and Dicer-CKO$_{MG}$. **q:** Sashimi plot of the region around exon 23 which is deleted in the Dicer-CKO[1]. In the Dicer-CKO$_{MG}$, 19 out of 32 reads skip exon 23 showing a 60% deletion. Statistics: mean ± S.D., Independent Samples T-Test and Levene’s Test for Equality of Variances, 2-tailed, significant differences are indicated, ***: p < 0.0001.

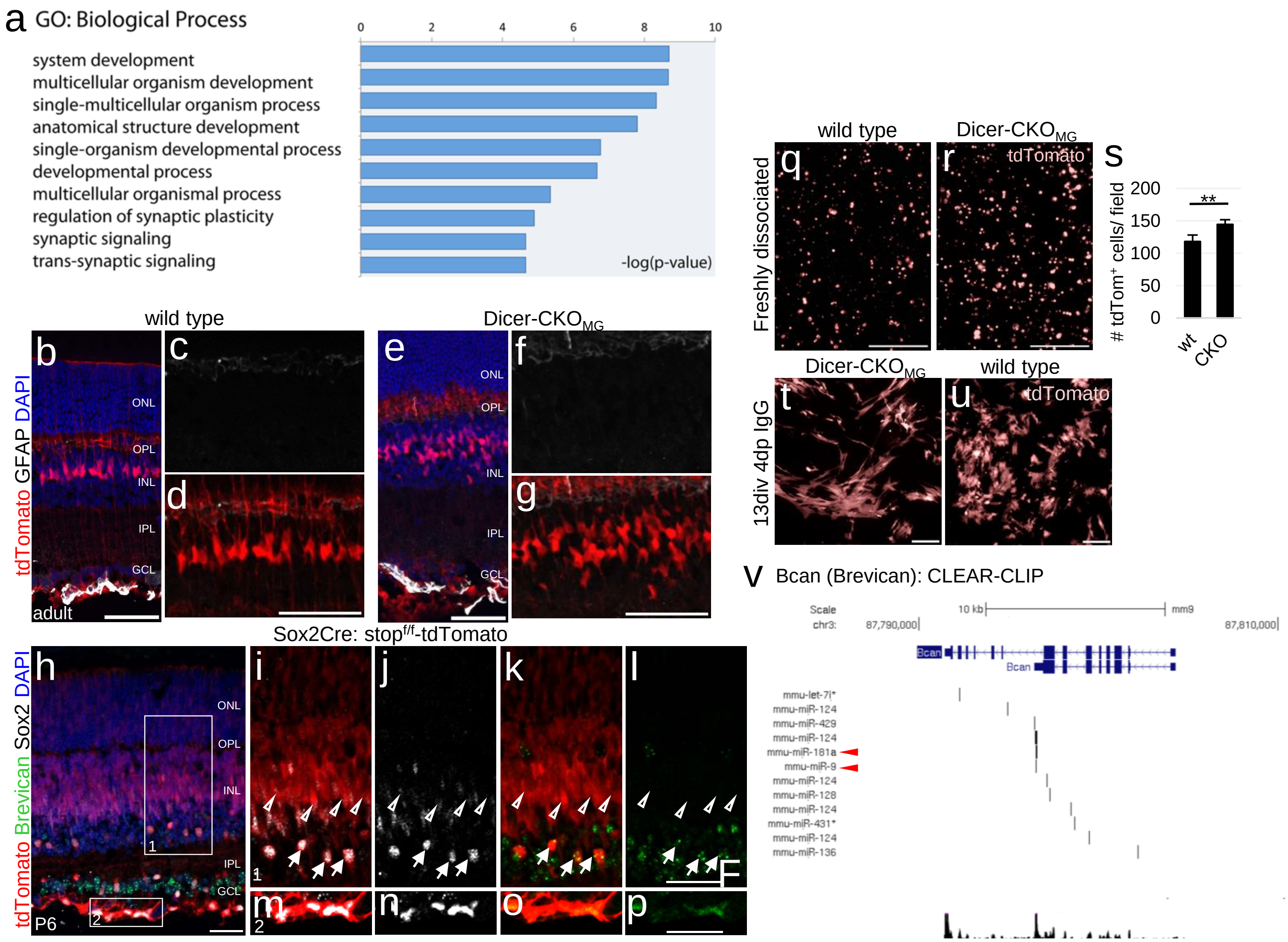

Supplementary Figure 6. Changes in gene expression in Müller glia of Dicer-CKO$_{MG}$ mice and analysis of adult Müller glia dissociated cell cultures

a: GO Profiler analysis of the 64 genes up-regulated (log2CMP > 5, log FC > 1) in the Dicer-CKO$_{MG}$ analyzed for enrichment in biological processes. One highly enriched Gene Ontology category, with 41/64 total up-regulated genes, was developmental process. **b-g:** Immunofluorescent labeling with antibodies against tdTomato and GFAP as well as DAPI nuclear staining in the wild type (b-d) and Dicer-CKO$_{MG}$ (e-g) retinal cross sections, one month after induction. **h-p:** Immunofluorescent labeling with antibodies against tdTomato, Brevican, Sox2 as well as DAPI nuclear staining in postnatal day (P) 6 retinal cross section of a Sox2CreER: stop$^{f/f}$-tdTomato mouse. At P6, Brevican was not found in in Sox2$^+$ immature MG (box 1, shown in i-l, unfilled arrowheads) but in Sox2$^+$ neurons in GCL (h) and the lower INL (box 1, shown in i-l, arrows), as well as in Sox2$^+$ astrocytes in the NFL (box 2 in m-p). **q-r:** Live images of freshly dissociated tdTomato$^+$ MG from adult wt (q) or Dicer-CKO$_{MG}$ retinas (r), on feeder layers of P12 MG. **s:** Number of tdTomato$^+$ MG per field in wt (5 individual mice) and Dicer-CKO$_{MG}$ mice (5 individual mice) in culture (one experiment). **t-u:** Live images of cultured tdTomato$^+$ MG from adult Dicer-CKO$_{MG}$ (t) and wild type mice (u) treated with control antibodies. **v:** Snapshot of the Argonaute mRISC complex bound miRNAs targeting *Bcan* using the CLEAR-CLIP method (3) revealing the miRNAs bound to *Bcan* in P13 brain. Statistics: mean ± S.D., Independent Samples T-Test and Levene's Test for Equality of Variances, 2-tailed, Bonferroni-Holm correction, significant differences are indicated, **: p < 0.01. Scale bars in b, e, q-r, t-u: 100 µm in d, g: 50 µm, in h, l, p: 20 µm. MG: Müller glia, wt: wild type, ONL: outer nuclear layer, OPL: outer plexiform layer, INL: inner nuclear layer, IPL: inner plexiform layer, GCL: ganglion cell layer.

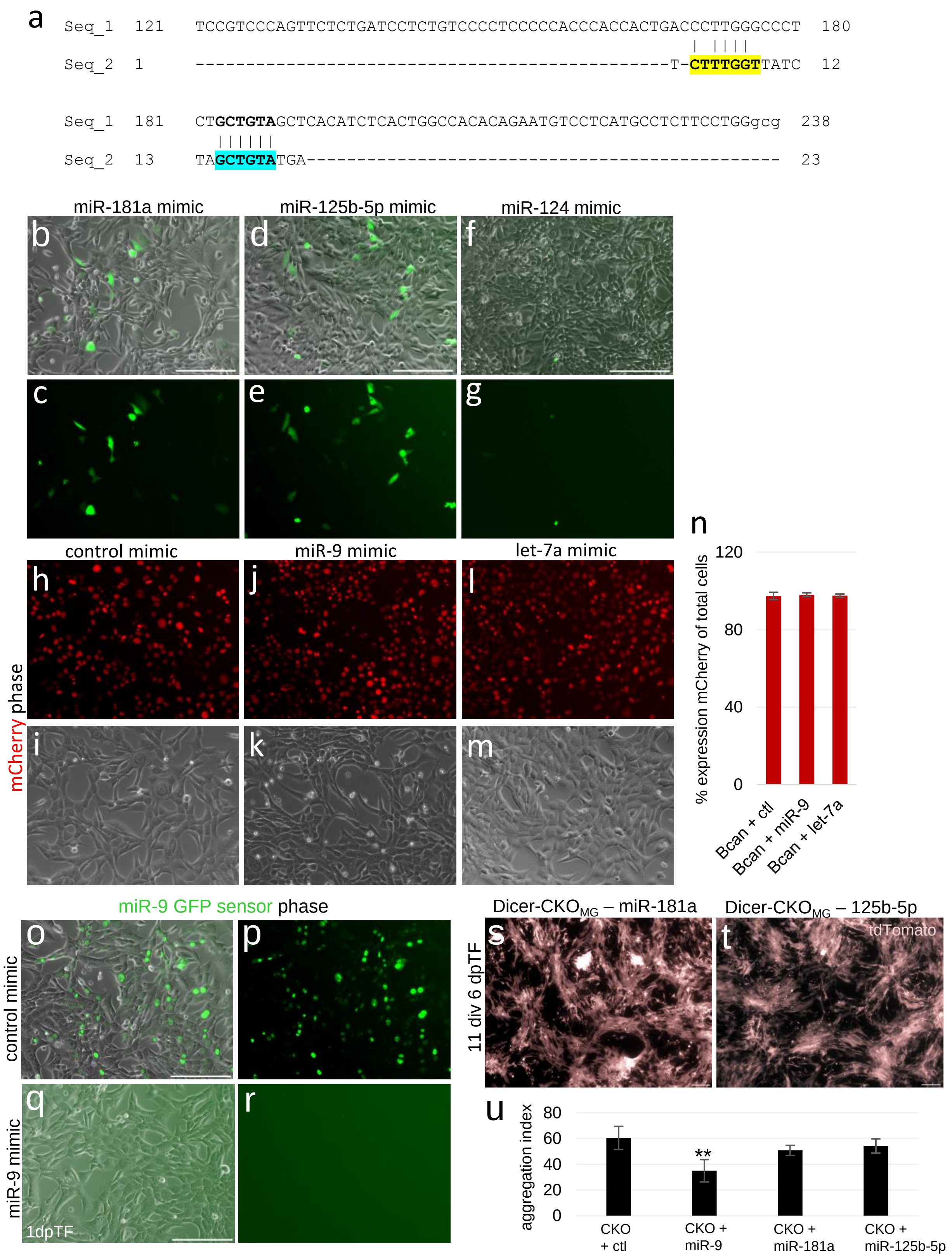

Supplementary Figure 7. Evidence for miR-9 regulation of Bcan and phenotype of Müller glia from Dicer-CKO$_{MG}$ mice

a: Alignment of miR-9 (sequence 2) in the *Bcan* 3'UTR region (sequence 1) in the *Bcan* GFP sensor for the short version of *Bcan*, which shows binding for the seed sequence (yellow) and another 6-mer (turquoise). **b-g:** Live images of 3T3 cells fibroblasts transfected with the *Bcan* 3'UTR sensor (plasmid) and either miR-181a (b-c), miR-125b-5p (d-e) or miR-124 mimics (f-g). **h-m:** Live images of 3T3 cells fibroblasts transfected with mCherry transfection control for cultures co-transfected with either control, miR-9, or let-7a mimics. **n:** Quantification of the number mCherry$^+$ cells of total number of cells. **o-r:** Live images of 3T3 fibroblasts transfected with mir-9 GFP sensor and either control mimics (o-p) or miR-9 mimics (q-r, experiment was performed 3 times). **s-t:** Live images of cultured tdTomato$^+$ MG from adult Dicer-CKO$_{MG}$ mice transfected with miR-181a (s), or miR-125b-5p (t). **u:** Quantification of the aggregation (see Methods) in control miR, miR-9, miR-181a, or miR-125b-5p transfected Dicer-CKO$_{MG}$ cultures (n = 4 individual mice per condition). Statistics: mean ± SD, Independent Samples T-Test and Levene's Test for Equality of Variances, 2-tailed, Bonferroni-Holm correction, significant differences are indicated, **: p = 0.01. Scale bars in b, d, f, o, q: 100 µm, s-t: 200 µm.

Supplementary Table 1: miRWalk 2.0 predictions for of the 41 genes that increase >2 fold in the Dicer-CKO$_{MG}$ for the five most highly expressed Müller glia miRNAs

miR-204	miR-125b-5p	miR-9	miR-181a	let-7
Arc	Arc	Arc	Atf3	Arc
Atf3	Atf3	Atf3	Cebpb	Atf3
Cebpb	Bcan	**Bcan**	Col27a1	Bcan
Col27a1	Csrnp1	Cebpb	Csf1r	C1qb
Csf1r	Cx3cr1	Col27a1	Csrnp1	C1qc
Csrnp1	Ddr2	Col27a1	Ddr2	Col27a1
Cst3	Egr3	Csf1r	Egr2	Csf1r
Cx3cr1	Faim2	Csrnp1	Eln	Csrnp1
Ddit3	Fosl2	Cx3cr1	Faim2	Cst3
Ddr2	Hk2	Ddr2	Fosl2	Cx3cr1
Egr2	Irf1	Faim2	Gadd45b	Ddr2
Egr3	Lmna	Fosl2	Hk2	Egr2
Eln	Mafb	Gadd45b	Irf1	Egr3
Faim2	Maff	Hk2	Lmna	Eln
Fosl2	Mafk	Irf1	Mafb	Faim2
Hk2	Nnat	Lmna	Mafk	Fosl2
Irf1	Parvb	Mafb	Map1b	Gadd45b
Lmna	Rgs9	Maff	Nnat	Hexb
Mafb	Sgk1	Mafk	P2ry12	Hk2
Maff	Slc17a7	Map1b	Parvb	Irf1
Mafk	Snap25	Nnat	Rgs9	Lmna
Map1b	Socs3	P2ry12	Sgk1	Mafb
Nnat	Stx3	Parvb	Slc12a5	Maff
P2ry12	Stxbp1	Rgs9	Socs3	Mafk
Parvb	Tnfaip3	Robo3	Stx3	Map1b
Rgs9		Scg2		Nnat
Robo3		Slc12a5		P2ry12
Scg2		Slc17a7		Parvb
Slc12a5		Socs3		Rgs9
Slc17a7		Stx3		Robo3
Snap25		Stxbp1		Scg2
Socs3		Tnfaip3		Sgk1
Stx3				Slc12a5
Stxbp1				Slc17a7
Tnfaip3				Snap25
				Socs3
				Stx3
				Stxbp1
				Tnfaip3

Supplementary Table 2. Glial genes in Dicer-CKO$_{MG}$ in the RNA-Seq

Gene Symbol	description	DicerCKO normLogCPM	wt normLogCPM	Increase in Cko vs wt	Dicer.raw	wt.raw
Aqp4	aquaporin 4	10.79	10.76	0.03	27887	37735
Gfap	glial fibrillary acidic protein	5.21	4.38	0.8	582	640
Glul	glutamate-ammonia ligase (glutamine synthetase)	14.95	15.28	-0.33	497777	865470
Rlbp1	retinaldehyde binding protein 1	11.88	12.57	-0.69	59381	132888
Slc1a3	solute carrier family 1 (glial high affinity glutamate transporter), member 3	11.32	11.83	-0.51	40287	79258
Sox2	SRY (sex determining region Y)-box 2	8.63	9.01	-0.37	6252	11213
Sox9	SRY (sex determining region Y)-box 9	9.55	8.92	0.63	11837	10589
Vim	vimentin	9.22	10.02	-0.8	9380	22650

Supplementary Table 3: primary antibodies

**antibody**	**concentration**	**Company, Catalog #**
rat anti RFP (tdTomato)	1:500	Antibodies online, ABIN334653
mouse anti glutamine synthetase (GS)	1:200	Millipore, MAB 302
rabbit anti Sox9	1:1000	Millipore, AB5535
goat anti Sox2 (Y-17)	1:100	Santa Cruz, sc-17320
rabbit anti Brevican (*Bcan*)	1:200	Novus, NBP1-89992
rabbit anti GFAP	1:1000	Dako, Z033401-2
rabbit anti caspase3	1:100	BD Pharmingen, 559565
mouse anti PKC	1:200	Sigma, P5704, clone MC5
goat anti Otx2	1:200	R&D Systems, BAF1979
chicken anti GFP	1:500	Abcam,

Supplementary Table 4: mimics

name	sequence	Catalog number
miRIDIAN microRNA mouse mmu-miR-9-5p mimic	UCUUUGGUUAUCUAGCUGUAUGA	C-310402-07-0005
miRIDIAN microRNA mouse mmu-miR-124-3p mimic	UAAGGCACGCGGUGAAUGCC	C-310390-05-0005
miRIDIAN microRNA mouse mmu-miR-181a-5p mimic	AACAUUCAACGCUGUCGGUGAGU	C-310435-05-0005
miRIDIAN microRNA mouse mmu-miR-125b-5p mimic	UCCCUGAGACCCUAACUUGUGA	C-310393-05-0050
miRIDIAN microRNA mouse mmu-let-7a-5p mimic	UGAGGUAGUAGGUUGUAUAGUU	C-310503-07-0005
miRIDIAN microRNA mimic negative control	Negative control sequences based on C. elegans microRNAs have minimal sequence identity in human, mouse, and rat.	CN-001000-01-50

Supplementary Table 5: RT-qPCR primers

Gene name	Forward sequence (5' to 3')	Reverse sequence (3' to 5')
Actb (beta actin)	GGCTGTATTCCCCTCCATCG	CCAGTTGGTAACAATGCCATGT
Bcan	TGCGCGTCAAGGTAAACGAA	GACCCCGGAATCATTGGGC
Maff	AGGGGTTCTCAGAGAGCCAG	AGGGGTTCTCAGAGAGCCAG
Egr3	CCGGTGACCATGAGCAGTTT	TAATGGGCTACCGAGTCGCT
Atf3	GAGGATTTTGCTAACCTGACACC	TTGACGGTAACTGACTCCAGC

Supplementary Table 6: RT-qPCR primers for miRNAs

miRNA	Forward sequence (5' to 3')	Reverse sequence (3' to 5')	Stem loop RT primer (3' to 5')
miR-204	CGC GCT TCC CTT TGT CAT CC	TCG GAG CGC CAA GTG TAC AG	TGA GCA GCG CTC GGA GCG CCA AGT GTA CAG GCG CTG CTC AAG GCA TAG
miR-125b-5p	CCG CTC CCT GAG ACC CTA A	CGA AGG AAC TTG GGA TAT GAC G	ATA GGG GCG CGA AGG AAC TTG GGA TAT GAC GCG CCC CTA TTC ACA AGT
miR-9	GCC GGT CTT TGG TTA TCT AGC	TTT CCT CGA CCG CAC CAC TC	AAA TCG CAG CTT TCC TCG ACC GCA CCA CTC GCT GCG ATT TTC ATA CAG
miR-181b	GCG CCA ACA TTC ATT GCT GTC	GAT CAA GGG GCA GCA CGT AG	GGC TAG CAG CGA TCA AGG GGC AGC ACG TAG GCT GCT AGC CAC CCA CCG
let-7c	CGG CCT TGA GGT AGT AGG TT	GCT TCG CTC TTA TTT CCT GAT GG	CGC TAC AGG CGC TTC GCT CTT ATT TCC TGA TGG GCG CGA TGT CAA CCA TAC
5s	GCC ATA CCA CCC TGA ACG	TGC AGG GTC CGA GGT ATT CG	ACT GCT GCG CTG CAG GGT CCG AGG TAT TCG GCG CAG CAG TAA AGC CTA
