# Supplementary material for: Müller glial microRNAs are required for the maintenance of glial homeostasis and retinal architecture
Source: Nat Commun. 2017 Nov 17;8:1603. doi: 10.1038/s41467-017-01624-y (PMC5693933; doi:10.1038/s41467-017-01624-y)
Supplement: Supplementary file 3 — Description of Additional Supplementary Files [file 41467_2017_1624_MOESM3_ESM.pdf]

File Name: Supplementary Data 1

Description: Each sheet in the Excel file contains raw data used to create the figure for which the sheet is named, e.g. the sheet titled “Figure 1w” has data for Figure 1w.
